# Rate and breadth of protein evolution are only weakly correlated

**DOI:** 10.1186/1745-6150-7-8

**Published:** 2012-02-15

**Authors:** Sergey A Naumenko, Alexey S Kondrashov

**Affiliations:** 1 Institute for Information Transmission Problems, Russian Academy of Sciences, Bolshoi Karetny pereulok 19, Moscow 127994, Russia; 2 Department of Bioengineering and Bioinformatics, M. V. Lomonosov Moscow State University, Vorbyevy Gory 1-73, Moscow 119992, Russia; 3 Life Sciences Institute and Department of Ecology and Evolutionary Biology, University of Michigan, Ann Arbor, Michigan 48109, USA

## Abstract

**Background:**

Evolution at a protein site can be characterized from two different perspectives, by its rate and by the breadth of the set of acceptable amino acids.

**Results:**

There is a weak positive correlation between rates and breadths of evolution, both across individual amino acid sites and across proteins.

**Conclusions:**

Rate and breadth are two distinct, and only weakly correlated, characteristics of protein evolution. The most likely explanation of their positive correlation is heterogeneity of selective constraint, such that less functionally important sites evolve faster and can accept more amino acids.

**Reviewers:**

This article was reviewed by Eugene V. Koonin, Arcady R. Mushegyan, and Eugene I. Shakhnovich.

## Background

Evolution of a protein, at a particular site, can be characterized from two different perspectives [1,2]. The first is the rate of evolution, defined as the number of amino acid replacements which occur per unit of astronomical time or per time required for one selectively neutral substitution. The second is the "breadth" of evolution, *i. e*., the diversity of acceptable amino acids, which can be defined as the probability that two independent replacements of a particular amino acid at a site lead to the same amino acid.

*A priori*, one could expect the rate and the breadth of protein evolution to be positively correlated across sites. Indeed, let us assume, in the spirit of the neutral theory [3], that, at a site, some amino acids are permitted, and confer the same fitness, and other amino acids are forbidden, because they confer very low fitnesses. Then, a site with a large number of permitted amino acids should display both a high rate and a wide breadth of evolution. Here, we investigate the rate-breadth correlation using the data on orthologous proteins from *Drosophila *and Mammalia.

## Methods

**Figure 1:**
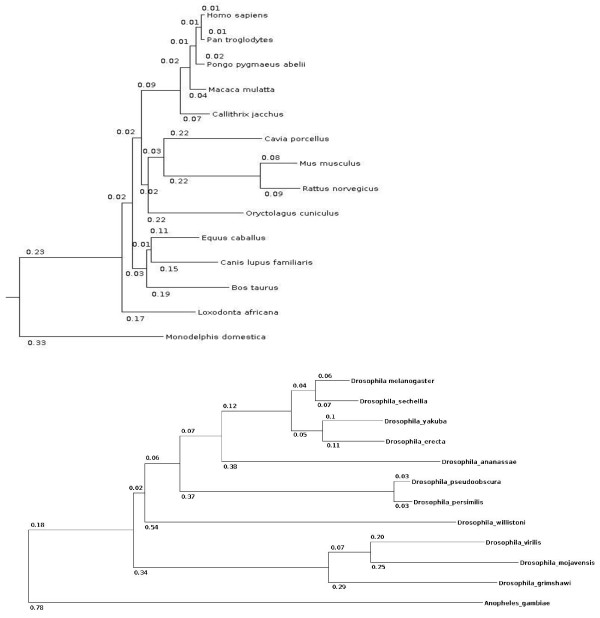
**Phylogenetic trees of placental mammals and of *Drosophila *flies used in our analysis**. Lengths of edges, measured in the units of K_s_, are shown.

We used two datasets: 13 genomes of placental mammals (all the available genomes with coverage above ×5) with *Monodelphis domestica *as an outgroup, and 11 genomes of species from genus *Drosophila *with *Anopheles gambiae *as an outgroup. Genome-size multiple alignments of 44 vertebrates and 13 insects were downloaded from UCSC Genome Browser database [4], and the subsets of genomes which we used were extracted from them. To obtain orthologous gene sets we extracted protein-coding sequences from whole-chromosome alignments using *H. sapiens *genome annotation for vertebrates and *D. melanogaster *genome annotation for insects. Alignments of orthologous genes were translated into amino acid sequences. Phylogenetic trees for the two datasets presented in the UCSC Genome Browser are reproduced in Figure 1.

To reconstruct substitutions on these phylogenetic trees, we used maximum likelihood method implemented in PAML software package [5]. For a multiple alignment of protein-coding genes translated into amino acid sequences, codeml command of PAML provides the list of amino-acid substitutions for each amino acid site.

The rate of evolution at a site was characterized by n, the number of independent substitutions of the common ancestral amino acid. Thus, with N species in the dataset, the rate can vary between 0 and N. The breadth B of acceptable amino acids at a site was characterized by a quantity which is analogous to virtual heterozygosity, widely used in population genetics,

(1) $$B=\left[n/\left(n-1\right)\right]\left[1-\overset{k}{\underset{i=1}{\sum }}p_{i}^{2}\right]$$

where k is the number of different amino acids with which the ancestral amino acid was substituted at the site, and *p_i _*is the frequency of the i-th amino acid. B provides an unbiased sample estimate of the effective number of acceptable amino acids at a site, because it can be interpreted as the fraction of pairs of substitutions of the ancestral amino acid where the new amino acids are different from each other. Indeed, this fraction can be expressed as

(2) $$\left[n\left(n-1\right)/2-\overset{k}{\underset{i=1}{\sum }}x_{i}\left(x_{i}-1\right)/2\right]/\left[n\left(n-1\right)/2\right]$$

where *x_i _*is the number of substitutions of the ancestral amino acid with the i-th amino acid. It is easy to show that expression (2) is equal to B, because $\overset{k}{\underset{i=1}{\sum }}x_{i}=\text{n}$ and *p_i _*= *x_i _*/n.

B can vary between 0 (all the observed substitutions lead to the same amino acid, implying that only 1 amino acid is acceptable as a substitution) and 1 (all the observed substitutions lead to different amino acids, implying an infinite variety of acceptable substitutions). Obviously, B can be calculated only for sites where at least 2 substitutions were observed. A measure of amino acid diversity which is equivalent to B be has been proposed in [6].

## Results

**Figure 2:**
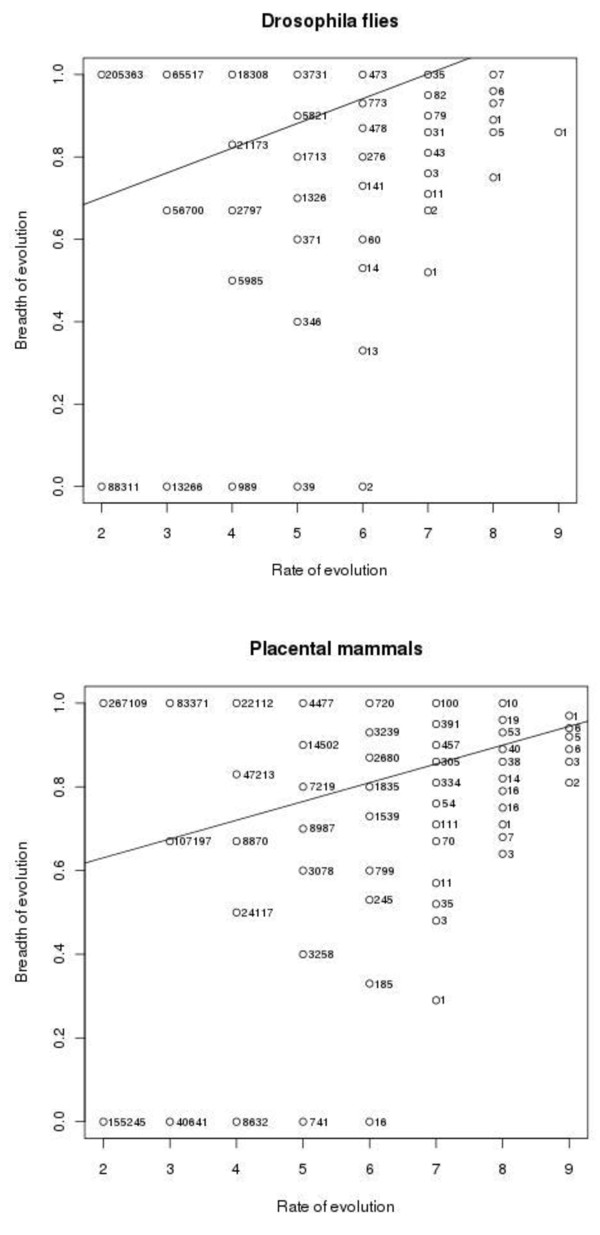
**Numbers of sites with different combinations of values of n and B in *Drosophila *flies (left) and placental mammals (right)**.

**Figure 3:**
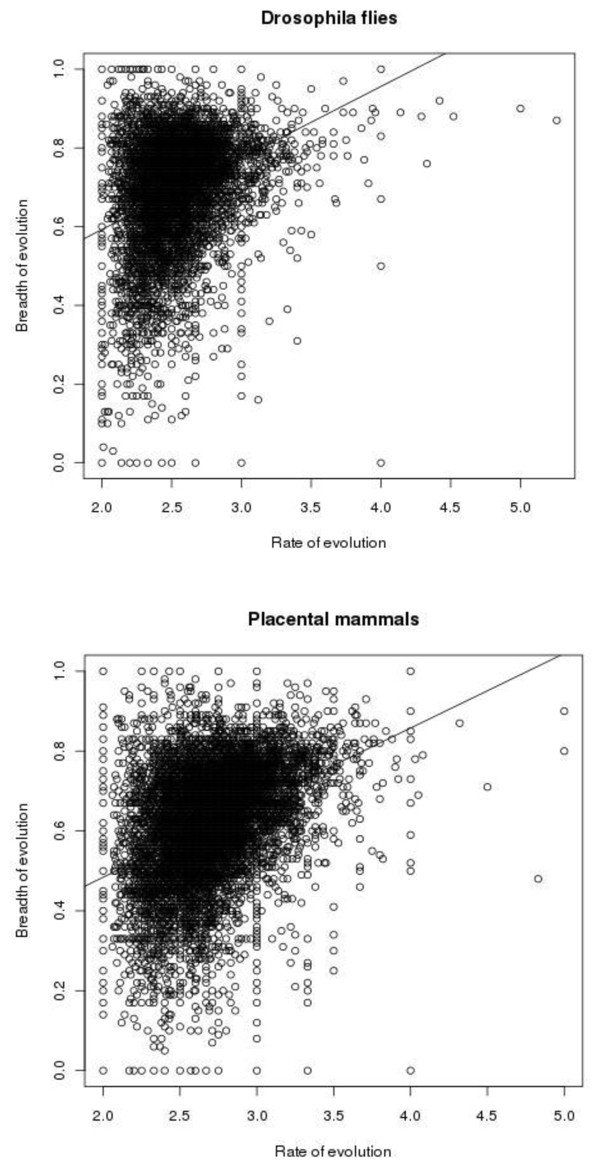
**Rates and breadths of evolution of all proteins in *Drosophila *flies (left) and placental mammals (right)**.

Figure 2 shows the relationship between the rate and the breadth of evolution at individual amino acid sites. There were also 2,886,411 and 324,330 sites with 0 and 1 substitution, respectively in *Drosophila *flies, and 5,086,661 and 353,544 sites with 0 and 1 substitution, respectively, in placental mammals, for which B cannot be calculated (not shown in Figure 2). Figure 3 displays the same relationship for whole proteins. For each protein, arithmetic means of n and B were calculated for all those sites where at least two substitutions of the ancestral amino acid occurred. Table 1 presents Pearson's and Spearman's correlation coefficients between the rate and the breadth of evolution, calculated from these data. All these coefficients are significantly different from 0 (p-value < 2.2e-16).

**Table 1:** Correlation coefficients between the rate and the breads of evolution for 20 amino acids

	*Drosophila *flies		Placental mammals	
	**Pearsons' R**	**Spearman's rho**	**Pearsons' R**	**Spearman's rho**

Over sites	0.12	-0.11	0.11	-0.12

Over proteins	0.29	0.26	0.34	0.39

**Figure 4:**
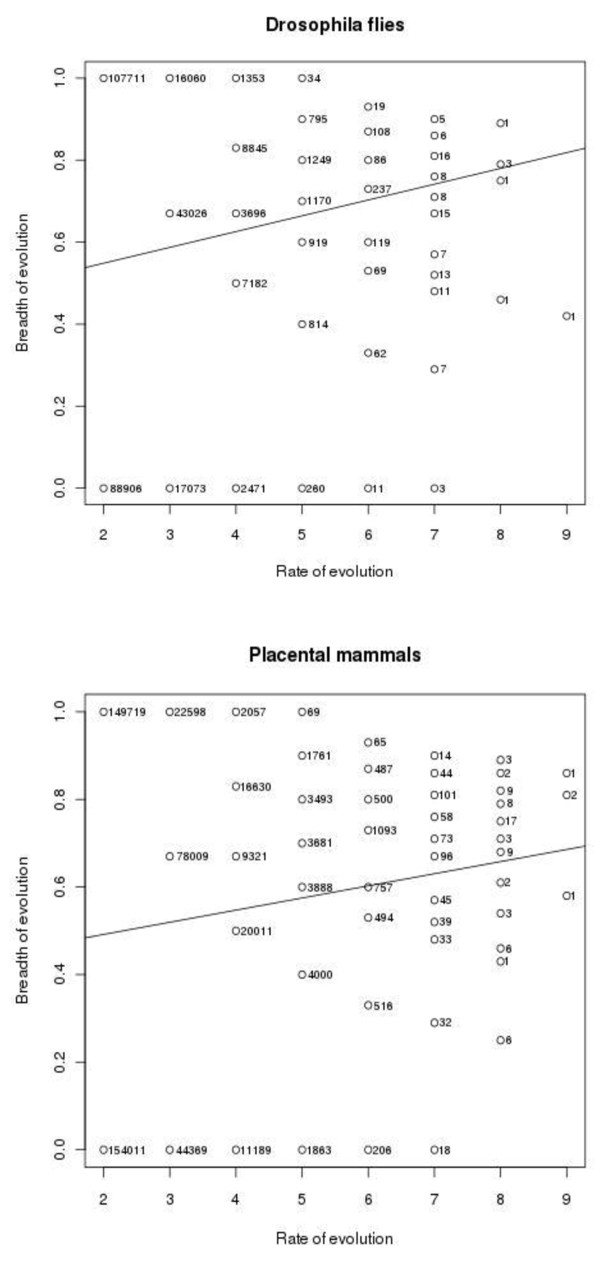
**Numbers of sites with different combinations of values of n and B in *Drosophila *flies (left) and placental mammals (right) calculated using classes of amino acids**.

**Figure 5:**
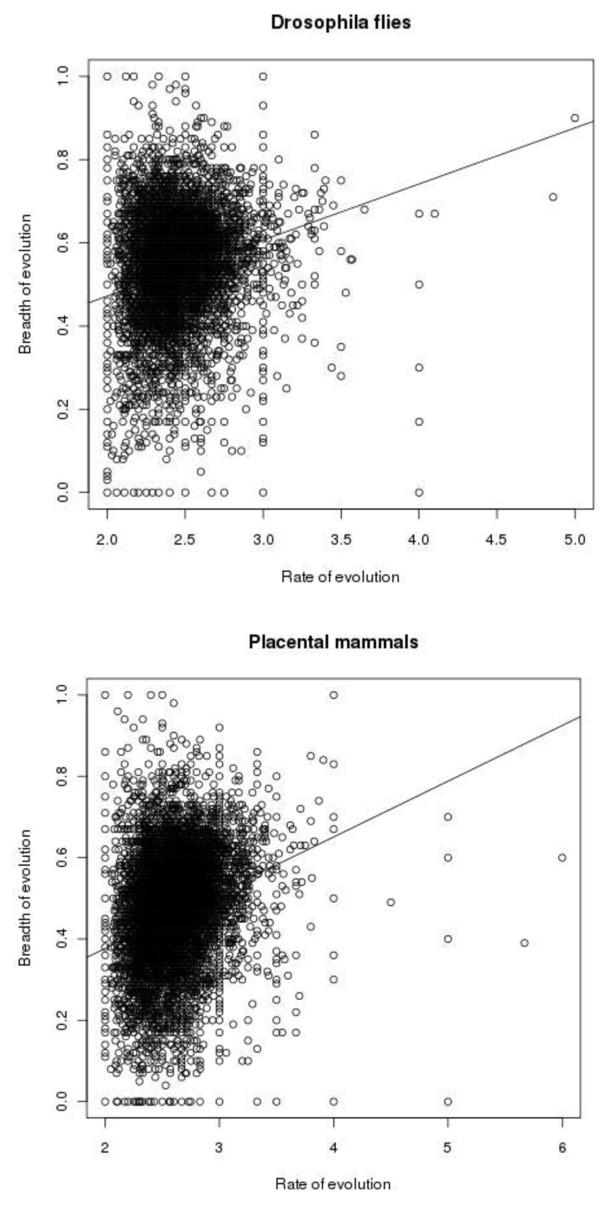
**Rates and breadths of evolution of all proteins in *Drosophila *flies (left) and placental mammals (right) calculated using classes of amino acids**.

Figures 4 and 5, and Table 2 present analogous data for amino acids grouped into 6 classes, according to [7] (AVLIMC, FWYH, STNQ, KR, DE, GP), such that only those substitutions that involve amino acids from different classes were taken into account. All the coefficients of correlation presented in Table 2 are significantly different from 0 (p-value < 2.2e-16).

**Table 2:** Correlation coefficients between the rate and the breads of evolution for 6 classes of amino acids

	*Drosophila *flies		Placental mammals	
	**Pearsons' R**	**Spearman's rho**	**Pearsons' R**	**Spearman's rho**

Over sites	0.06	-0.08	0.06	-0.05

Over proteins	0.19	0.19	0.23	0.29

## Discussion

Our data demonstrate a weak positive correlation between the rate and the breadth of protein evolution across both individual sites and whole proteins (Table 1). Indeed, very weak negative Spearman's correlations across individual sites should probably be viewed as artifactual, because non-parametric rank correlation ignores the range of breadth values, which is much wider, and includes many 0 values, for low values of the rate of evolution (Figures 2 and 4). With this structure of data, a positive Pearson's correlation is consistent with a negative Spearman's correlation. Indeed, when data for whole proteins are considered, which do not possess this structure (Figures 3 and 5), Pearson's and Spearman's correlation coefficients are very close to each other.

Thus, at sites which evolve faster, the diversity of acceptable amino acids is also slightly higher. The simplest explanation of this positive correlation is that many amino acid replacements occur at selectively neutral, or nearly neutral, sites [3]. Such sites evolve at above-average rates, due to lack of selective constraint, and all amino acids are acceptable at them, leading to a positive rate-breadth correlation.

Still, this correlation is weak, at it is often the case in evolutionary bioinformatics [8,9]. In the case of individual sites, this must be partially due to a relatively small number of species in both datasets. Indeed, on a sample of only 11 or 13 genomes, the "real" values of rate and breadth of evolution are determined, for a particular site, only with substantial sampling errors, which reduces their correlation. However, this effect must be much less important when these characteristics are calculated for whole proteins, each consisting of many sites, and yet the correlation observed in this case is not much higher.

The overall lengths of phylogenetic trees, in the units of K_s_, are 3.20 and 2.08, for placental mammals and *Drosophila *flies, respectively. Thus, assuming that evolution at synonymous sites is approximately neutral, we can expect ~7 and ~4 nonsynonymous substitutions at a codon in these trees, as long as selective constraint is absent. Sites with that many substitutions are rare (Figure 2), indicating a widespread constraint on the rate of evolution. A value of breadth B at a site without constraint should be ~0.85, because an amino acid can have from 5 to 12 1-step neighbors in the genetic code table. Again, we mostly see lower values, indicating a substantial selective constraint in terms of the breadth, too.

Thus, the rate and the breadth are two different, and only weakly correlated, characteristics of protein evolution. Apparently, sophisticated evolution-based analyses of protein function [1,2] should take them into account separately. If an alignment of very many homologous proteins is available, so that these two characteristic can be ascertained, with a good precision, for individual sites, it may be interesting to study sites where the rate of evolution is high but the breadth is low, or *vice versa*. Sites which evolve very rapidly but accept only a small number of amino acids may experience multiple episodes of positive selection, and sites which evolve slowly but accept a wide variety of amino acids may be under epistatic selection, affected by evolution at other sites.

## Competing interests

The authors declare that they have no competing interests.

## Authors' contributions

Both authors planned the research, and SN was mostly responsible for carrying out the analysis. AK did most of the writing. Both authors read and approved the final manuscript.

## Reviewer's comments

Reviewer's report

Title: Rate and breadth of protein evolution are only weakly correlated

Version: 2 Date: 28 September 2011

Reviewer number: 1

Report form: Eugene V. Koonin

Review of "Rate and breadth of protein evolution are only weakly correlated" by Naumenko and Kondrashov

In this extremely brief manuscript, Naumenko and Kondrashov establish the connection between two characteristics of the evolution of amino acid sites in proteins: the rate of amino acid substitution and the range (breadth) of amino acid residues that are tolerated in the given position. A weak positive correlation between the two variables is demonstrated. This result is by no means unexpected, precisely because it seems intuitively highly plausible that 'less functionally important sites evolve faster and can accept more amino acids'. I nevertheless accept the authors' view that this connection cannot be taken for granted - indeed, it is possible to imagine a site at which two amino acids oscillate at a high rate - and thus is certainly worth documenting.

The manuscript, however, is fraught with a variety of conceptual, technical and presentational problems. At the level of general conclusions, my impression is that the authors more or less attempt to "have it both ways": the title for instance states that the two characteristics are 'only weakly correlated' but the interpretation mostly deals with the fact that positive correlation does exist. There is no real attempt to explain the modest magnitude of the correlation.

Furthermore, the reported observations are not put into a general context of the previous studies on connections between different evolutionary variables. For example, an obvious but potentially interesting parallel exists between the observations reported here and the previously described correlation between the evolutionary rate of an individual gene and its loss rate in the course of evolution [1,2]. In general, there is virtually no discussion in the manuscript and only 6 references, all of them purely technical. I am not at all suggesting that the number of references should be inflated artificially but this brevity is excessive and does not benefit the reader (to put it mildly).

Perhaps, most importantly, the extreme brevity of the manuscript results in the lack of sufficient detail on the methods and results; some of these details seem essential. We are not even told whether the reported correlations are R or R2 (a big difference). Furthermore, there are no p-values for these correlation coefficients. Without such statistical estimates, it is impossible to judge the quality and robustness of the results. At least to this reviewer, Figure 2 is a verystrange, counter-intuitive way to present the results. Figure 3 is easy to understand but this figure causes concerns of a different kind: could it be that the observed correlations are caused by outliers? The main, dense cloud of points seems pretty symmetrical in both A and B. I think it is important to address this issue. Nothing at all in this manuscript is described in sufficient detail -neither the sets of orthologous genes analyzed nor the alignment method not the tree construction procedure. This makes the work irreproducible and worse, impossible to assess objectively. My greatest concern perhaps is with the calculation of the diversity measure, B. I realize that by design this is an unbiased measure that is independent of the actual number of substitutions in a site (for n > 2). However, I am not sure that in practice there is no statistical bias toward less diversity in slow-evolving sites. Should that be the case, the result reported here simply would be an artefact. I believe that it is crucial for the authors to address this issue and more generally describe the methods adequately, even if they choose not to amend the other sections.

1. Krylov DM, Wolf YI, Rogozin IB, Koonin EV: Gene loss, protein sequence divergence, gene dispensability, expression level, and interactivity are correlated in eukaryotic evolution. Genome Res 2003, 13(10):2229-2235.

2. Wolf YI, Novichkov PS, Karev GP, Koonin EV, Lipman DJ: The universal distribution of evolutionary rates of genes and distinct characteristics of eukaryotic genes of different apparent ages. Proc Natl Acad Sci USA 2009, 106(18):7273-7280.

Quality of written English: Acceptable

**Response to Reviewer 1 **(Eugene V. Koonin)

At the level of general conclusions, my impression is that the authors more or less attempt to "have it both ways": the title for instance states that the two characteristics are 'only weakly correlated' but the interpretation mostly deals with the fact that positive correlation does exist. There is no real attempt to explain the modest magnitude of the correlation.

**We revised our discussion of the weak correlation observed. However, we did not try "to have it both ways", as we have no dog in this fight - instead, we just report a pattern which, apparently, has not been reported previously**.

Furthermore, the reported observations are not put into a general context of the previous studies on connections between different evolutionary variables. For example, an obvious but potentially interesting parallel exists between the observations reported here and the previously described correlation between the evolutionary rate of an individual gene and its loss rate in the course of evolution [1,2].

**This is reassuring: if Dr. Koonin could not think of anything closer to the pattern we report, our manuscript is definitely worth publishing... Which is strange, because a question we asked seems to be a rather basic one - and we were afraid that the answer is buried in the literature somewhere. We now cite **[1,2]** - although their relevance to our observation is rather indirect, to put it mildly**.

I am not at all suggesting that the number of references should be inflated artificially but this brevity is excessive and does not benefit the reader (to put it mildly).

**As this reviewer surely knows, Anton Chekhov regarded brevity as a "sister of talent". We now expanded our Discussion a little, mostly to reiterate that such a weak correlation seems puzzling to us. We do not have a definite explanation**.

Perhaps, most importantly, the extreme brevity of the manuscript results in the lack of sufficient detail on the methods and results; some of these details seem essential. We are not even told whether the reported correlations are R or R2 (a big difference). Furthermore, there are no p-values for these correlation coefficients. Without such statistical estimates, it is impossible to judge the quality and robustness of the results.

**We are thankful for this useful suggestion. Now we report both Pearson's andf Spearman's correlation coefficients, which, due to huge sample sizes, are all significantly different from zero, despite being rather small**.

At least to this reviewer, Figure 2 is a very strange, counter-intuitive way to present the results.

**Well, but how else could we present these data? Both the abscissa and the ordinate can accept only a small number of values (for the ordinate, these values are different for different abscissa values). We tried our best**.

Figure 3 is easy to understand but this figure causes concerns of a different kind: could it be that the observed correlations are caused by outliers? The main, dense cloud of points seems pretty symmetrical in both A and B. I think it is important to address this issue.

**There is nothing in the data which suggests that outliers are disproportionally important**.

Nothing at all in this manuscript is described in sufficient detail -neither the sets of orthologous genes analyzed nor the alignment method not the tree construction procedure.

**We did not use any special tree construction procedure. As stated in the manuscript, we simply reproduced the already-published phylogenies from the UCSC site. Also, as stated in the manuscript, we used standard alignments, fully described in the references provided. We only used PAML and calculated correlation coefficients for its outputs, as described**.

This makes the work irreproducible and worse, impossible to assess objectively.

**We cannot agree - one can take the same standard alignments we used and easily get the same results - as long as we did not make any stupid mistakes**.

My greatest concern perhaps is with the calculation of the diversity measure, B. I realize that by design this is an unbiased measure that is independent of the actual number of substitutions in a site (for n > 2). However, I am not sure that in practice there is no statistical bias toward less

diversity in slow-evolving sites.

**We are not sure what to do about this comment. As stated in our manuscript, B (which is not novel - it has already been introduced, for a different purpose, in Ref. 6) is the proportion of pairs of novel amino acids, substituting the ancestral amino acid, which consist of different amino acids. Thus, B must be unbiased, as far as the number of observed substitutions is concerned, and is is not clear what is a statistical bias "in practice". We would love to fix something here - but do not see what is broken**.

Reviewer's report

Title: Rate and breadth of protein evolution are only weakly correlated

Version: 2 Date: 5 October 2011

Reviewer number: 2

Report form:

The authors demonstrate that the frequency of amino acid substitutions (rate of protein sequence evolution) and the repertoire of replacements in the substituted sites (breadth of protein sequence evolution) are correlated only weakly.

I was curious to see, however, that the data indicate something more than that.

Namely, there is a strong depletion not only of "high rate/low breadth", but "high rate/medium to medium-high breadth" categories of sites and proteins. Is it just the artefact of small number of fast-evolving sites in these species, or is anything else going on? One way to partially test this would be to examine the data for viruses, where there are not that many proteins, but much more lineages and substitutions.

I would also like to comment on the null hypothesis. The authors state in the Background that "A priori, it is not obvious how the rate and the breadth are correlated across sites and proteins", but in the rest of the study seem to express mild surprise that the correlation between rate and breadth is so weak. I feel that expecting little if any correlation would be sensible, because this would involve fewer "just so" stories than otherwise. On the other hand, if most sites turn out to be constrained as they appear to be, this is evidence in favor of different possible kinds of selection, as the authors indicate. Can they also indicate whether this bears in any way on the various neutral models of protein evolution?

Quality of written English: Acceptable

**Response to Reviewer 2 **(Arcady R. Mushegyan)

There is a strong depletion not only of "high rate/low breadth", but "high rate/medium to medium-high breadth" categories of sites and proteins. Is it just the artefact of small number of fast-evolving sites in these species, or is anything else going on? One way to partially test this would be to examine the data for viruses, where there are not that many proteins, but much more lineages and substitutions.

**An interesting observation. Indeed, viruses or mitochondria, with very fat multiple alignemnts (but only a few of them), are definitely worth looking at. However, we feel that they should be treated separately, because rather different analyses may be needed**.

I would also like to comment on the null hypothesis. The authors state in the Background that "A priori, it is not obvious how the rate and the breadth are correlated across sites and proteins", but in the rest of the study seem to express mild surprise that the correlation between rate and breadth is so weak. I feel that expecting little if any correlation would be sensible, because this would involve fewer "just so" stories than otherwise. On the other hand, if most sites turn out to be constrained as they appear to be, this is evidence in favor of different possible kinds of selection, as the authors indicate. Can they also indicate whether this bears in any way on the various neutral models of protein evolution?

**Essentially the same point has been made by reviewer 3. We now think that it is reasonable to treat a substantial positive correlation as "null-hypothesis", because it is expected in the simplest case of neutrality with some strong selective constraint. We revised the text accordingly**.

Reviewer's report

Title: Rate and breadth of protein evolution are only weakly correlated

Version: 2 Date: 1 November 2011

Reviewer number: 3

Report form:

Review of the paper Rate and breadth of protein evolution are only weakly correlated'' by SA Naumenko and AS Kondrashov.

In this paper Naumenko and Kondrashov study the relationship between evolutionary rate at a protein locus and ''evolutionary breadth'', i.e. the diversity of amino acid substitutions at the same locus. The authors find very small correlation between the two quantities. The Bioinformatics analysis is carried out carefully and professionally and the data itself is interesting. However, I have several concerns about discussion of the results and most important, their relevance.

1) What did the authors expect in the first place and why? What kind of model did they have in mind? Are their results surprising or expected/trivial?

2) Due to stability constraints buried amino acids are much more conserved (i.e. evolve much slower and/or have less diverse substitutions) as shown in our papers in 1999 (JMB, v.291, pp.177-96) and in 2001 (ibid, v.312, pp, 289-307).

To that end this study can be significantly deepened and extended if protein sites are classified by their buriedness (e.g. solvent accessibility, ASA or related measure) and proper comparison is made. Further the authors should make themselves familiar with recent work by Francosa and Xia, Mol Biol Evol v.26, pp.2387-95 (2009) and Wilke et al Genetics v.188, p.479 (2011) which address the issue of relation of evolutionary rate to location of amino acid in structure.

3) The author's definition of ''breadth'' does not consider the proximity of physical-chemical properties of amino acids. For example one can observe many mutations of V to, say L in a hydrophobic core but these are almost neutral as amino acids are very similar in many respects. One way to address this shortcoming is to use any substitution matrix and weight diversity definition accordingly. Alternatively the authors can group amino acids by their properties (e.g one such grouping is presented in the 1999 JMB mentioned above) and consider amino acid changes only occurring between groups and disregard changes within same group. The results of such analysis might be much more informative, especially compared with ''raw'' data presented here. It will provide an insight of what kind of pressure (to maintain stability, function or specific or promiscuous protein-protein interactions) is most manifest in observed evolutionary patterns.

Eugene Shakhnovich Harvard UniversityQuality of written English: Acceptable

**Response to Reviewer 3 **(Eugene I. Shakhnovich)

1) What did the authors expect in the first place and why? What kind of model did they have in mind? Are their results surprising or expected/trivial?

**We expected a stronger correlation. Indeed, in the simplest case, some amino acids are permitted at a site (under selective neutrality) and some are forbidden (under strong negative selection) - which would lead to a strong correlation. We now state this - not as a part of our personal intellectual histories, but as a reasonable *a priori *assumption**.

2) Due to stability constraints buried amino acids are much more conserved (i.e. evolve much slower and/or have less diverse substitutions) as shown in our papers in 1999 (JMB, v.291, pp.177-196) and in 2001 (ibid, v.312, pp, 289-307). To that end this study can be significantly deepened and extended if protein sites are classified by their buriedness (e.g. solvent accessibility, ASA or related measure) and proper comparison is made....

**We agree. However, this sounds like a separate project, investigating the phenomenon we describe here**.

3) The author's definition of ''breadth'' does not consider the proximity of physical-chemical properties of amino acids....

**Following this suggestion, we now grouped amino-acids into 6 classes and repeated our analysis. The results are included in the paper. Essentially, nothing changed**.

1 Eugene V. Koonin Second review (response to the authors' rebuttal) of "Rate and breadth of protein evolution are only weakly correlated" by Naumenko and Kondrashov The revised manuscript is an improvement over the original version, in my view, primarily because the authors now clearly state their null hypothesis in the Background section (in response to the comments of reviewers #2 and #3). However, I found that other aspects of the manuscript problems and in particular some of the responses to my original comments remain problematic.

From the rebuttal:

We now cite [1,2] - although their relevance to our observation is rather indirect, to put it mildly.

Reviewers' response: I take exception to this and continue to maintain that the relevance is substantial and clear. These references investigate the relationship between evolutionary rates at different scales, essentially like the work of Naumenko and Kondrashov, although different measures of long-term evolutionary rates were employed.

Moreover, the citation itself is disingenuous: "Still, this correlation is weak, at it is often the case in evolutionary bioinformatics [8,9]". This was not at all the context in which I expected these references to be cited, the connection is substantial (see above) rather than purely formal as the quoted sentence implies. Moreover, although the statement about weak correlations is generally reasonable, the cited papers actually report relatively strong correlations. This is not at all an adequate response to a reviewer's comment. If the authors actually do not see the relevance, they are entitled to their opinion and could simply say that much; the reader would know of the dissenting view of the reviwer from the comment. However, if they choose to cite these references in the main body of the paper, I believe this should be done properly.

From the rebuttal:

As this reviewer surely knows, Anton Chekhov regarded brevity as a "sister of talent". We now expanded our Discussion a little, mostly to reiterate that such a weak correlation seems puzzling to us. We do not have a definite explanation.

Reviewer's response: I still maintain that the manuscript is too brief and does not include either proper background or sufficient discussion. I am not sure the Chekhov quote (indeed, popular among Russian-reading literati) really belongs here. But, if the authors wish to head in that direction, I am afraid the irony is on them. Apparently, Chekhov's formula (from a letter to his brother) is a paraphrase of a line from Shakespeare's Hamlet: 2 '...brevity is the soul of wit' which is pronounced by Lord Polonius to announce Hamlet's purported madness to the King and Queen http://shakespeare.mit.edu/hamlet/full.html. That character is not necessarily the best example to follow.

From the rebuttal:

We are thankful for this useful suggestion. Now we report both Pearson's andf Spearman's correlation coefficients, which, due to huge sample sizes, are all significantly different from zero, despite being rather small.

Reviewer's response: Certainly, it is good that the authors have followed the suggestion to include p-values. However, I am still troubled by the negative Spearman coefficient values for individual sites, the authors attempt on explanation in the beginning of the Discussion notwithstanding. These negative values are of about the same magnitude as the corresponding positive values of the Pearson correlation coefficient and in my view are problematic.

Reviewer's report

Title: Rate and breadth of protein evolution are only weakly correlated

Version: 5 Date: 23 December 2011

Reviewer number: 2

Report form:

Nothing further

Quality of written English: Acceptable
